# Supplementary material for: An ultrafast diamond nonlinear photonic sensor
Source: Nat Commun. 2025 Sep 25;16:8300. doi: 10.1038/s41467-025-63936-8 (PMC12462469; doi:10.1038/s41467-025-63936-8)
Supplement: Supplementary file 1 — Supplementary Information [file 41467_2025_63936_MOESM1_ESM.pdf]

Supplementary Information

An ultrafast diamond nonlinear photonic sensor

Daisuke Sato[1†], Junjie Guo[1†], Takuto Ichikawa[1], Dwi Prananto[2], Toshu An[2], Paul Fons[3], Shoji Yoshida[1], Hidemi Shigekawa[1] & Muneaki Hase[1★]

[1]*Department of Applied Physics, Faculty of Pure and Applied Sciences, University of Tsukuba, 1-1-1 Tennodai, Tsukuba 305-8573, Japan.*

[2]*School of Materials Science, Japan Advanced Institute of Science and Technology, Nomi, Ishikawa 923-1292, Japan.*

[3]*Department of Electronics and Electrical Engineering, Faculty of Science and Technology, Keio University, 3-14-1 Hiyoshi, Kohoku-ku, Yokohama, 223-8522, Kanagawa, Japan.*

[†]These authors equally contributed to this work.

[★]Correspondence and requests for materials should be addressed to M. H. (mhase@bk.tsukuba.ac.jp).

Supplementary Note 1. Energy level diagrams of NV^- center in diamond.

The measurement of the electric field is based on the linear electro-optic effect (i.e., the Pockels effect; the linear variation of the optical index of refraction upon application of quasi-static electric field)[1], which is a second-order nonlinear optical effect. Since the electro-optic effect is basically induced by non-resonant transitions, it does not require a real charge carrier excitation. That means our 1.5 eV photon ($\hbar\boldsymbol{\omega}$) does not directly interact with the NV^- spin states[2] as schematically shown in Fig. S1.

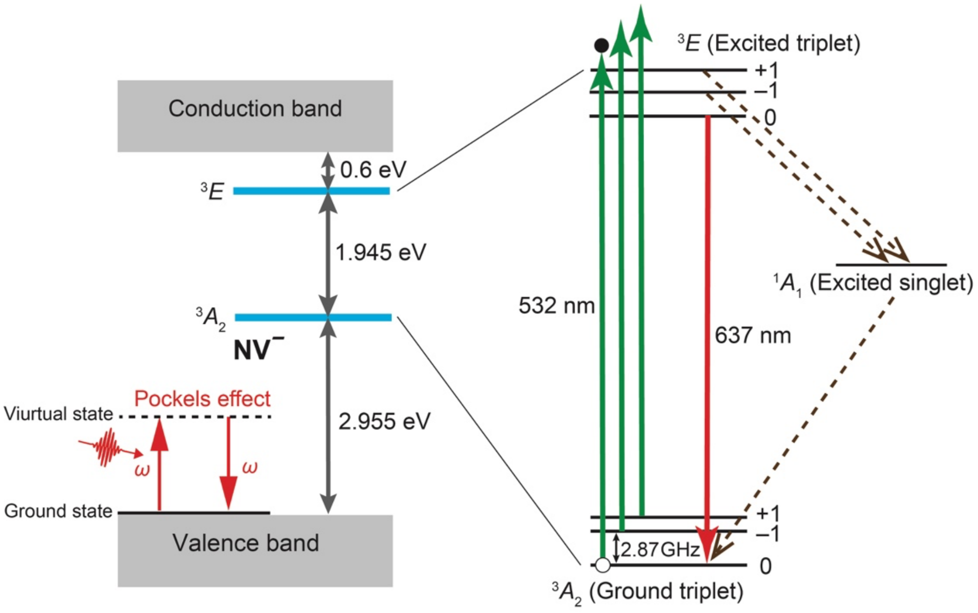

Figure S1: The energy level diagram around the NV^- center in diamond. The 1.5 eV photon ($\hbar\omega$) used in the present study does not interact with the NV^- spin states directly, but non-resonant transitions occur from the ground state to/from a virtual state, corresponding to the Pockels effect; $\chi_{ij}^{(2)}(\omega = \omega - 0)$.

Supplementary Note 2. The charge states of NV centers

We show the density of NV^- state was a maximum at a N^+ ion dose of 1×10^{12} cm^{-2} based on Optically Detected Magnetic Resonance (ODMR) measurements, which is shown in Fig. S2 below. Thus, the ODMR results indicate the optimal density for the enhancement of the EO effect observed in the previous study is coincident with the maximum NV^- density[3].

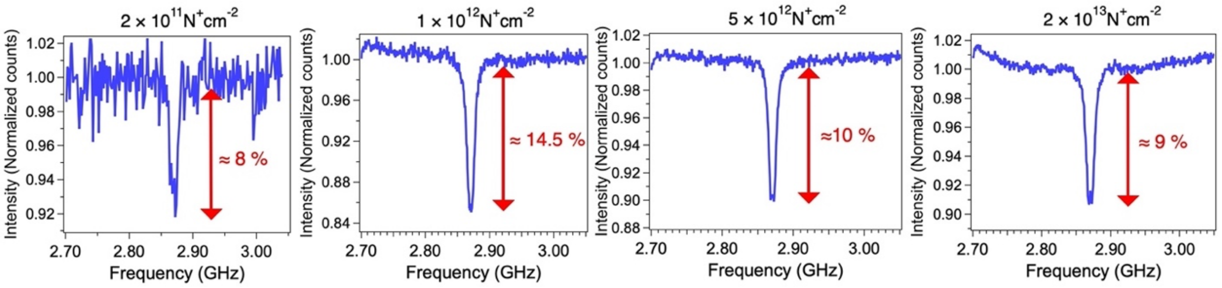

Figure S2: The ODMR spectra obtained at room temperature for the NV diamond samples using 532 nm cw laser. Adapted from the Supplementary Information in Ref. [3]. The ODMR measurements show the contrast of the NV^- resonant dip was maximized at the dose of 1×10^{12} N^+ cm^{-2}, indicating the density of NV^- was enhanced at 1×10^{12} N^+ cm^{-2}.

Supplementary Note 3. The estimation of the diameter of NV center ensemble.

From the fluorescence imaging in Fig. 2b, the size of the NV center ensemble is estimated as shown in Fig. S3. Based on a Gaussian fit of the line profile near the center, the spatial resolution of the diamond NV probe was found to be better than ≈ 660 nm and even potentially ≤ 500 nm because of the enhancement of the EO sensitivity at the apex of the NV tip: the $^{14}N^+$ ion dose at the most intense red-color region ≈ 500 nm in Fig. 2b (and Fig. S3) is the same as the optimal density of 1×10^{12} cm^{-2} found in the previous study[2].

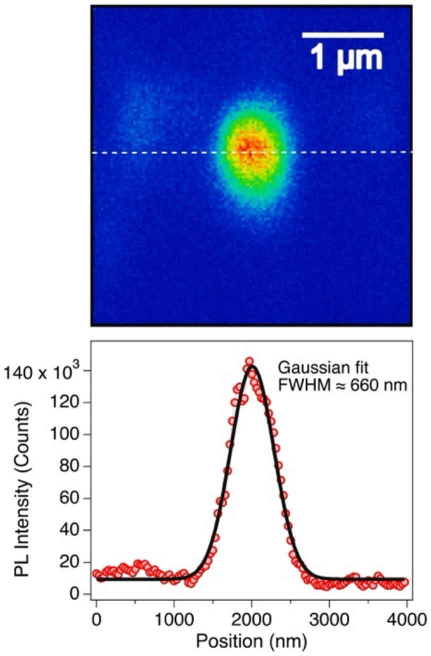

Figure S3: The photoluminescence (PL) image (Top) and the line profile (Bottom) at the horizontal dashed line. Based on the Gaussian fit of the profile, the spatial resolution of the diamond NV probe was better than ≈660 nm and potentially ≤500 nm due to enhancement of the EO sensitivity at the apex of the NV tip.

Supplementary Note 4. The electro-optic (EO) sampling technique.

The refractive index under a quasi-static electric field E accompanying the Pockels and Kerr electro-effect is given by,

$$n(E) = n_0 - \frac{1}{2} n_0^3 r^{ij} E + n_2 E^2, \quad \text{(S1)}$$

where n_0 is the static refractive index, r^{ij} and n_2 are the Pockels and Kerr coefficients, respectively. Using the general relations of $n_2 = \frac{3}{4 n_0^2 \epsilon_0\, c} \chi_{ijk}^{(3)}$ and $r^{ij} = -\frac{2}{n_0^4} \chi_{ij}^{(2)}$ (Ref. [1]), we have

$$n(E) = n_0 + \frac{1}{n_0} \chi_{ij}^{(2)} E + \frac{3}{4 n_0^2 \epsilon_0\, c} \chi_{ijk}^{(3)} E^2. \quad \text{(S2)}$$

In the case of the NV center introduced diamond, in which inversion symmetry is broken, we have $\chi_{ij}^{(2)} \neq 0$ and $\chi_{ijk}^{(3)} \neq 0$, so $\Delta n(E)$ is the sum of the two terms, whereas in the case of other quantum defects, such as SiV centers, $\chi_{ij}^{(2)} = 0$ and $\chi_{ijk}^{(3)} \neq 0$, so $n(E)$ is given only by the third-order term, which is generally smaller than the second-order term. The 13-fold enhancement of the EO response observed in the NV diamond can thus be explained by the additional second-order term because of $\chi_{ij}^{(2)} \neq 0$ (Refs. [3], [4]).

To measure the electro-optic (or anisotropic reflectivity) $\Delta R_{eo}(t)/R_0$ signal, the photo-current from the two Si-PIN photodetectors was subtracted and then amplified by current amplifier as shown in Fig. S4. In the experiment, we measure the anisotropic reflectivity change $\Delta R_{eo}(t)$ as the EO signal[5,6],

$$\frac{\Delta R_{eo}(t)}{R_0} = \frac{4}{n_0^2 - 1} \left(\Delta n_x(t) - \Delta n_y(t) \right) = \frac{4 n_0^3}{(n_0^2 - 1)} r^{ij} \Delta E(t) = \frac{-8}{(n_0^2 - 1) n_0} \chi_{ij}^{(2)} \Delta E(t), \quad \text{(S3)}$$

where $n_x(t) = n_0 + \frac{1}{2} n_0^3 r^{ij} E$ and $n_y(t) = n_0 - \frac{1}{2} n_0^3 r^{ij} E$ are the x- and y-components of the indices of refraction, and R_0 is the reflectivity without photoexcitation. Eq. (S3) clearly indicates the EO sampling is a measure of the change of the electric field $\Delta E(t)$ and this is only possible when $\chi_{ij}^{(2)} \neq 0$, corresponding to the case of the NV-containing diamond.

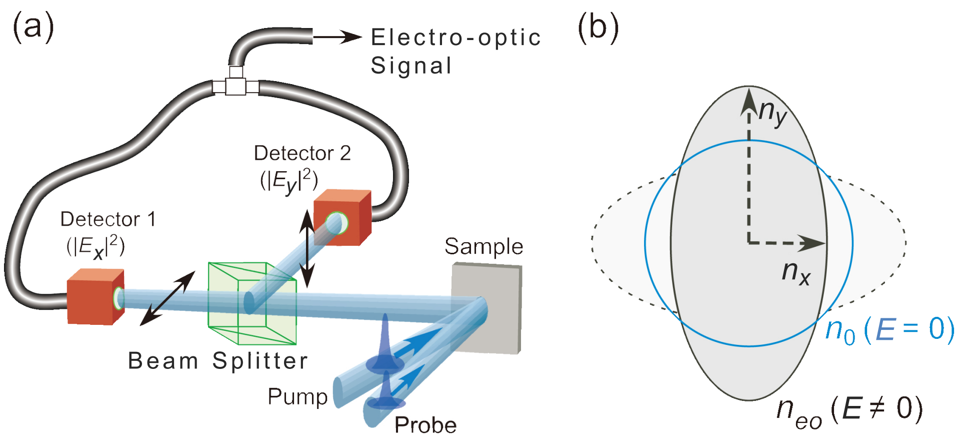

Figure S4: (a) Electro-optic detection of an anisotropic change in the refractive index. Adapted from Ref. [6]. The polarization of the probe was 45° with respect to the optical plane. (b) The isotropic refractive index (n_0) before the photoexcitation ($\boldsymbol{E}$ = 0) and the anisotropic refractive index (n_{eo}) after the photoexcitation ($\boldsymbol{E} \neq 0$).

Supplementary Note 5. Setups for pump-probe EO sampling and AFM systems.

The optical path of all optical components for the $\Delta R_{eo}/R_0$ measurement is shown in Fig. S5, and is a well-established experimental technique in the field of ultrafast laser spectroscopy[5-8]. We have built a homemade microscopy system combined with a self-sensing AFM system, as schematically shown in Fig. S6 and as photograph in Fig. S7.

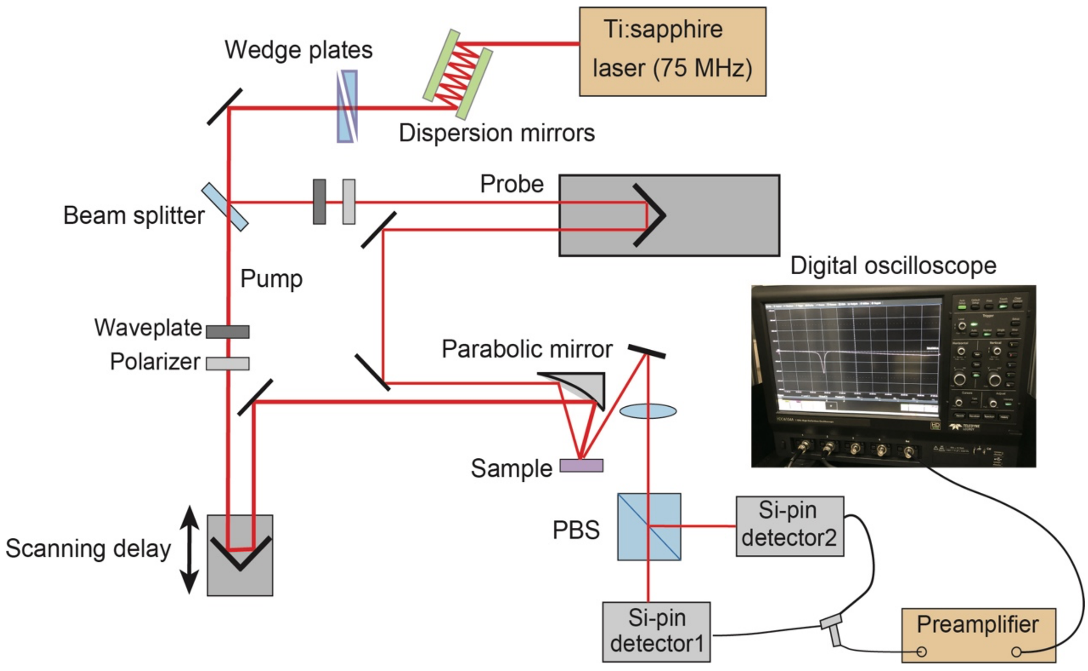

Figure S5: Time-resolved pump-probe electro-optic (EO) sampling setup using a 10-fs pulsed laser. The time delay between the pump and probe pulses was scanned by using an oscillating mirror. Both pump and probe beams were focused onto the sample by a parabolic mirror, and only reflected probe was detected and amplified to obtain the $\Delta R_{eo}/R_0$ signal.

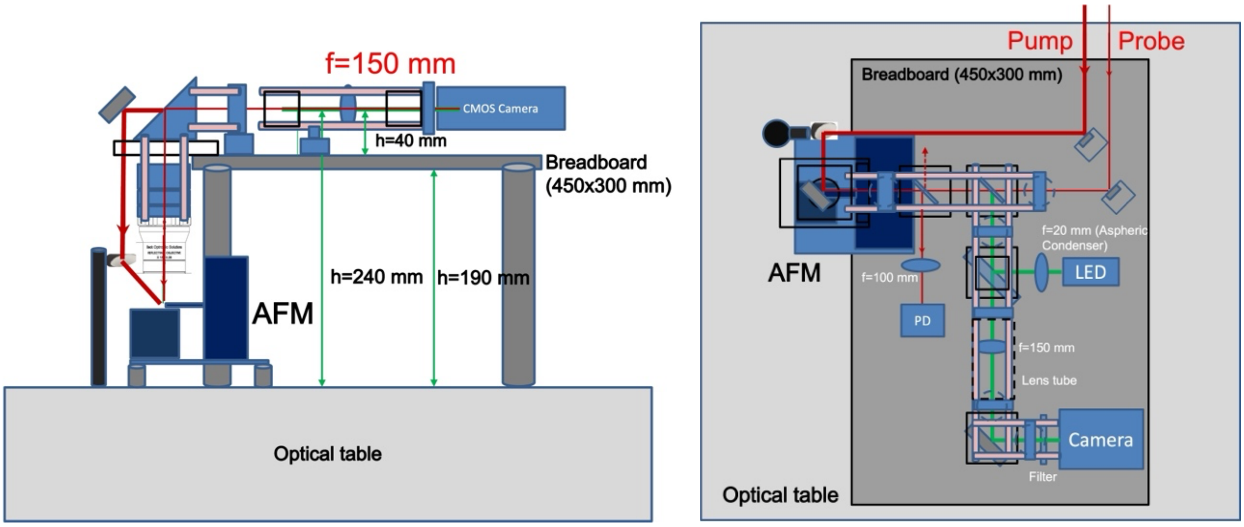

Figure S6:. Schematic of the AFM system designed on the breadboard. (Left) Side view. (Right) Top view.

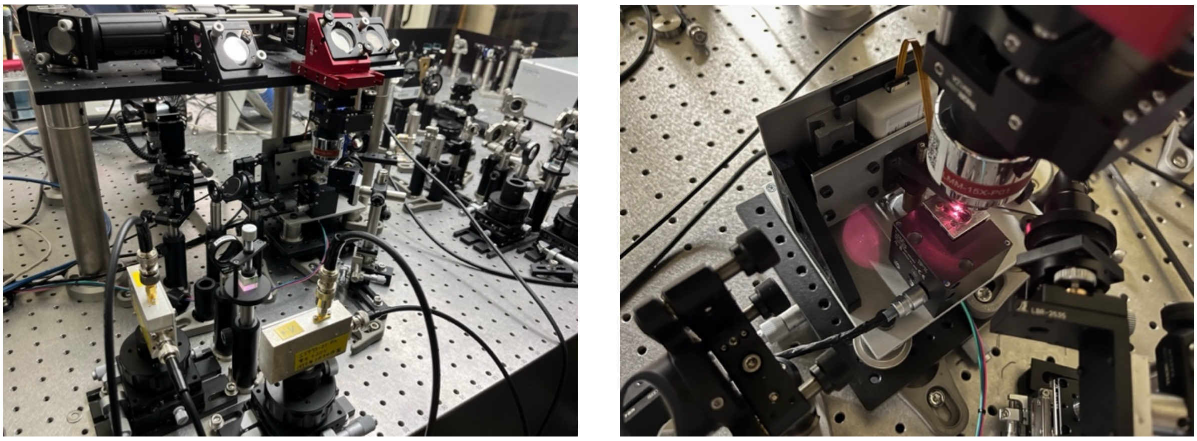

Figure S7:. (Left) Photograph of our AFM system, designed in Fig. S6. (Right) The enlarged photo of sample holder and piezo scanner under the illumination of both pump and probe beams.

Supplementary Note 6. The value of electro-optic (Pockels) coefficient r_{ij} of NV diamond.

Regarding to Fig. 3, Fig. 3b was measured without NV probe, therefore, the Pockels coefficient should be for n-GaAs ($r_{41} \approx -1.6$ pm V^{-1}), while in the case of Fig. 3c, we measure the EO response from n-GaAs through the NV ptrobe, that means the Pockels coefficient should be used for NV diamond, which is in genaral unkown. We recently, however, obtained the value of $\chi_{ij}^{(2)}$ for NV diamond using a second harmonic generation approach as described in Ref. [4], that was $\chi_{ij}^{(2)} \approx 100$ pm V^{-1}. Using the general relationship of $r^{ij} = -\frac{2}{n_0^4}\chi_{ij}^{(2)}$(Ref. [1]), we obtain $r^{ij} \approx -6$ pm V^{-1} for $n_0 = 2.4$ (diamond). Note that in general $|r^{33}| > |r^{22}|$ for $3m(C_{3v})$ crystal

symmetry (NV diamond) and r^{33} may play a central role, but more theoretical and experimental work is required to fully understand the Pockels coefficient of the NV diamond.

Supplementary Note 7. Raman spectra of the sample used.

The Raman spectra taken for 1ML and Bulk-WSe_2 are shown in Fig. S8. The 1ML-WSe_2 shows strong peak at 245 cm^{-1} together with a satellite peak at 254 cm^{-1}, whereas Bulk-WSe_2 shows weak signals at 245 cm^{-1} and 252 cm^{-1}, together with a small peak at 306 cm^{-1}, which indicate Raman signature as expected for 1ML and Bulk-WSe_2 (Refs. [9]-[11]), although a small redshift in the peak positions might originate from the effects of transfer process of WSe_2, e.g., carrier doping, surface oxidation, defects formation, and stress formation.

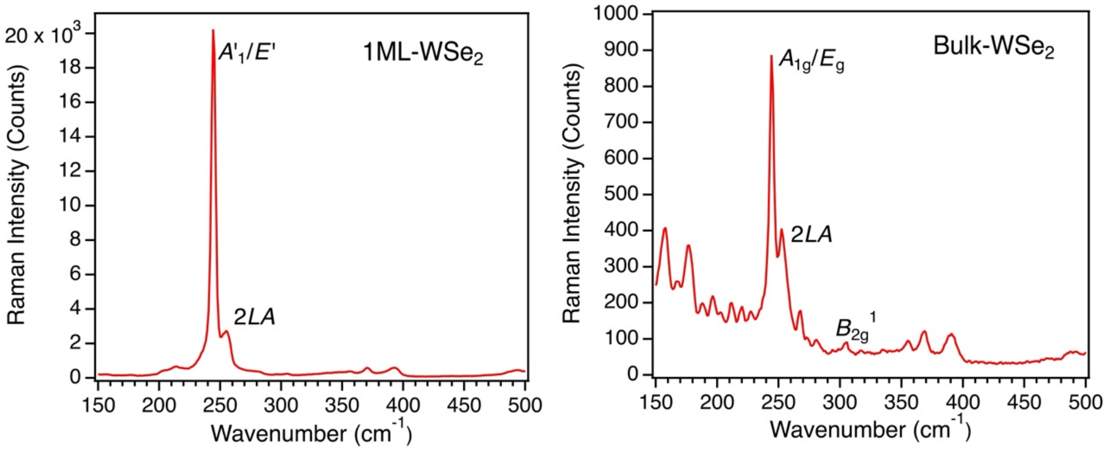

Figure S8:. Raman spectra obtained for 1ML-WSe_2 and Bulk-WSe_2 using a 532 nm laser at room temperature.

Supplementary Note 8. Dynamics of intraband and intervalley carrier relaxation.

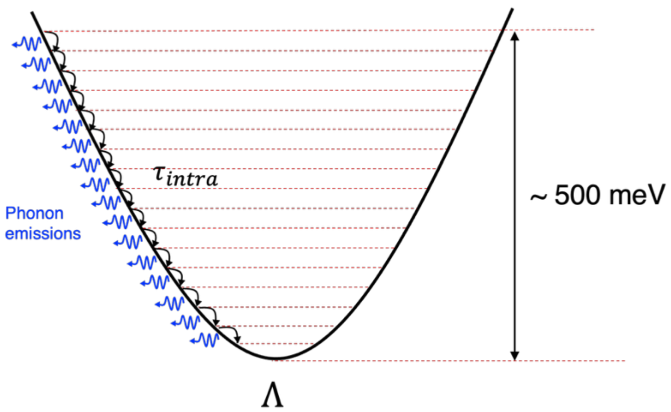

Figure S9: Schematic for intraband carrier relaxation via phonon emissions in bulk WSe_2. In the bulk WSe_2 the excited electrons decay via intraband (τ_{intra}) scattering at the Λ valley by emitting 16 optical phonons (7.5 THz ≈ 32 meV).

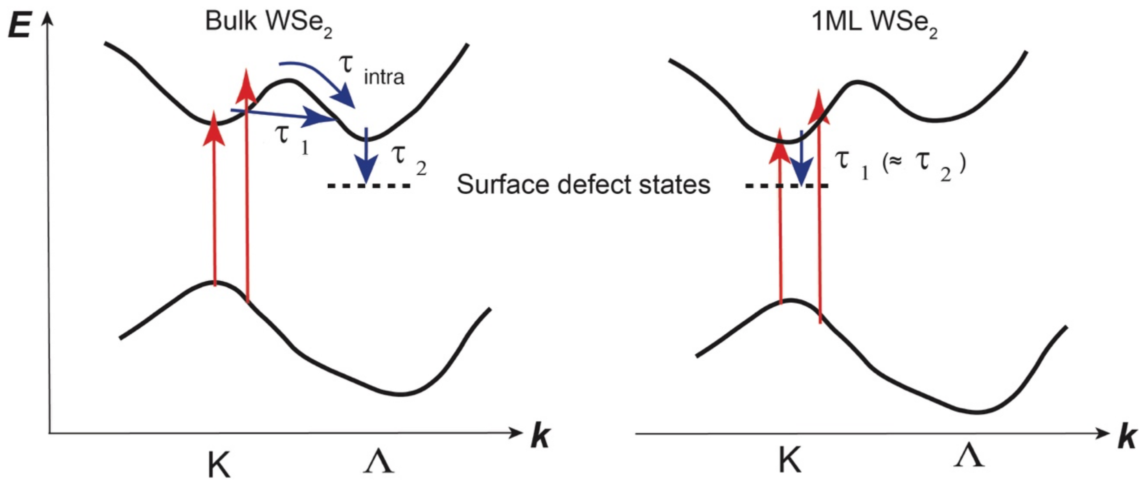

Figure S10: Schematic of carrier thermalization dynamics in bulk and 1ML WSe_2. (Left) In the bulk-WSe_2 the excited electrons initially decay via intra- (τ_{intra}) and inter-valley scattering from the K to Λ valley, followed by the trapping into surface defect states, resulting in double exponential relaxation with time constants of τ_1 and τ_2. (Right) In the 1ML-WSe_2 the excited electrons decay by the trapping into surface defect states at the K valley, resulting in single exponential relaxation with a time constants of τ_1.

Supplementary Note 9. Monte Carlo simulation (Stopping and Range of Ions in Matter: SRIM).

To assess how the NV-layer looks like, we have performed the Monte Carlo simulation using a popular code of Stopping and Range of Ions in Matter: SRIM[12]. As the results, the implanted nitrogen has an average depth of ~40 nm and is distributed like a Gaussian with a full width at half maximum (FWHM) of ~25 nm, as demonstrated in Fig. S11.

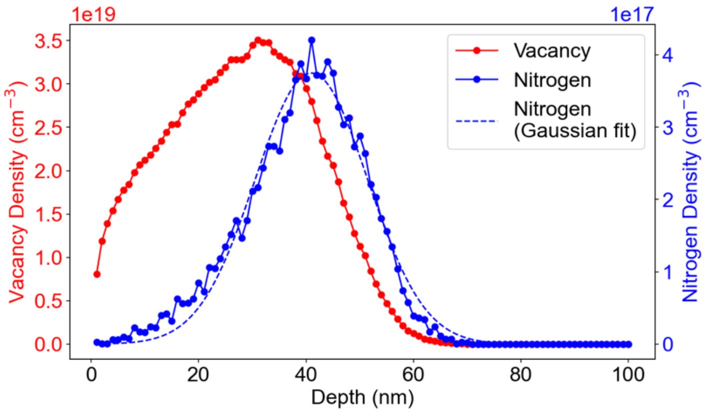

Figure S11: Distribution of nitrogen ions (blue dots) and vacancies (red dots) as a function of implantation depth, simulated by SRIM for the surface of (100) diamond for an implantation energy of 30 keV and an incident angle of 7°, with the fluence of 1×10^{12} ions cm^{-2}. The dashed line represents a fit using a Gaussian function.

Supplementary Note 10. Local structure of color centers in a diamond crystal.

Since pure defect-free diamonds are colorless and their crystal structure has spatial inversion symmetry, their second-order nonlinear susceptibility $\chi^{(2)}$ is strictly zero, and second-order nonlinear optical effects such as the Pockels effect do not occur[13-16]. To overcome this disadvantage, we have used diamond crystals with NV centers, where the second-order nonlinear susceptibility is non-zero ($\chi^{(2)} \neq 0$) due to the breaking of spatial inversion symmetry by the NV defects[4], and hence, the Pockels effect is expected to occur[3]. Note that other quantum defects in diamond, such as SiV (or GeV, etc.) centers[17], do not break the inversion symmetry (Fig. S12). Thus, the NV center is one of the color centers in diamonds that enables the EO sensing capability although other color centers, such as BV, OV centers, may also break the inversion symmetry[18].

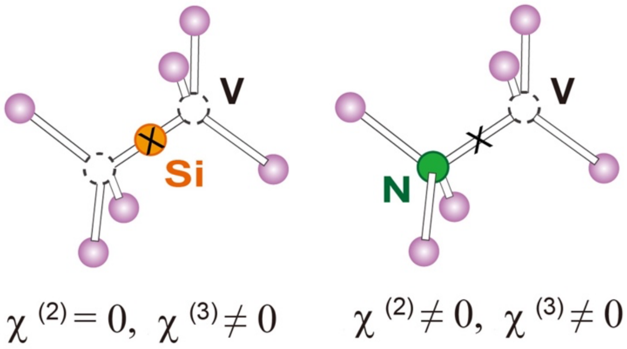

Figure S12: Local structure of color centers in a diamond crystal. In SiV (or GeV, SnV, PbV, etc.) centers, second-order nonlinear optical effects do not occur because the inversion symmetry is not broken, while in NV centers second-order nonlinear optical effects do occur due to the breaking of the inversion symmetry.

Supplementary Note 11. The force-distance curve of the NV tip.

To investigate if our NV tip was really approached on the sample surface (the height was quasi-zero), we took a force-distance curve for an n-GaAs wafer sample in air, as shown in Fig. S13. When using a commercial Si-tip from Sensor Tech Inc (PRSA-L300-F50-Si-PCB; Tip radius < 15 nm) the force-distance curve shows a snap into contact at ~100 nm and approached at T-B (resistance bridge; top minus bottom) = 35 mV, while in the case using the NV-tip it shows a snap into contact at ~180 nm and approached at T-B = 35 mV and when retracted it shows a jump out at ~ 420 nm. Thus, we can conclude that the NV tip was really approached on the sample surface.

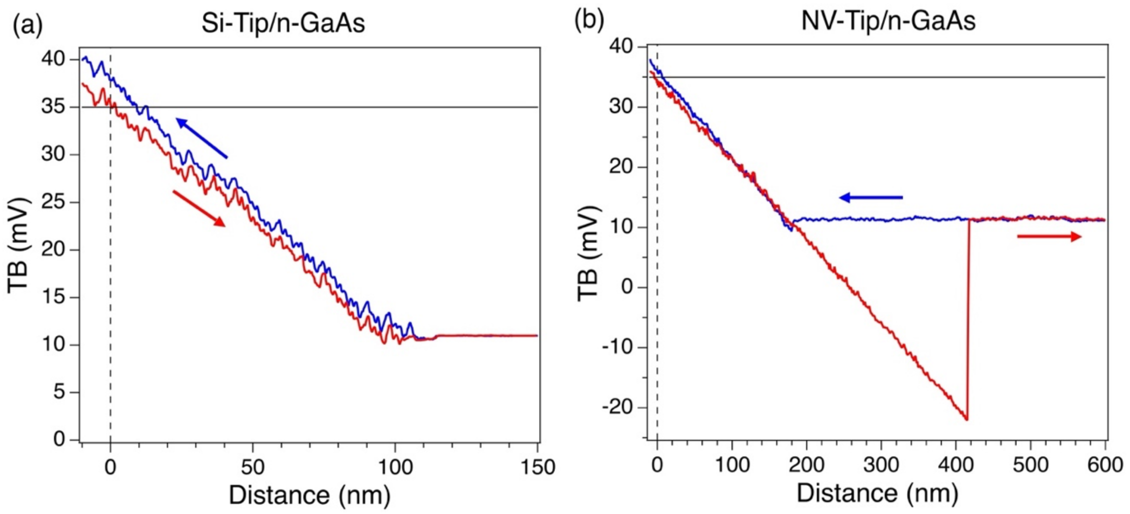

Figure S13: (a) The force-distance curve measured for Si-tip on an n-GaAs wafer. (b) The force-distance curve measured for NV-tip on an n-GaAs wafer. In both plots, T-B=35 mV corresponds to the approached point.

Supplementary References

[1] Boyd, R. W. *Nonlinear optics.* 3rd ed (Elsevier, 2003).

[2] Maze, J. R. *et al*. Nanoscale magnetic sensing with an individual electronic spin in diamond. *Nature* **455**, 644–647 (2008).

[3] Ichikawa, T., Guo, J., Fons, P., Prananto, D., An, T. & Hase, M. "Cooperative dynamic polaronic picture of diamond colour centres", *Nat. Commun.* **15**, 7174 (2024).

[4] Abulikemu, A., Kainuma, Y., An, T. & Hase. M. Second-harmonic generation in bulk diamond based on inversion symmetry breaking by color centers. *ACS Photon.* **8**, 988–993 (2021).

[5] Cho, G. C., Kütt, W. & Kurz, H. Subpicosecond time-resolved coherent-phonon oscillations in GaAs. *Phys. Rev. Lett*. **65**, 764–766 (1990).

[6] Hase, M., Katsuragawa, M., Constantinescu, A. M. & Petek, H. Coherent phonon-induced optical modulation in semiconductors at terahertz frequencies. *New J. Phys.* **15**, 055018 (2013).

[7] Hase, M., Kitajima, M., Constantinescu, A. M. & Petek, H. The birth of a quasiparticle observed in time-frequency space. *Nature* **426**, 51–54 (2003).

[8] Hase, M., Katsuragawa, M., Constantinescu, A. M. & Petek, H. Frequency comb generation at terahertz frequencies by coherent phonon excitation in silicon. *Nat. Photon.* **6**, 243–247 (2012).

[9] M. De Luca *et al.* New insights in the lattice dynamics of monolayers, bilayers, and trilayers of WSe_2 and unambiguous determination of few-layer-flakes' thickness. *2D Materials* **7**, 025004 (2020).

[10] Zhao, W. *et al*. Lattice dynamics in mono- and few-layer sheets of WS_2 and WSe_2. *Nanoscale* **5**, 9677 (2013).

[11] Terrones, H. *et al*. New first order Raman-active modes in few layered transition metal dichalcogenides. *Sci. Rep*. **4**, 4215 (2014).

[12] Ziegler, J. F., Ziegler, M. D. & Biersack, J. P. SRIM - the stopping and range of ions in matter. *Nucl. Instrum. Methods Phys. Res. Sect. B* **268**, 1818–1823 (2010).

[13] Aharonovich, I., Greentree, A. D. & Prawer, S. Diamond photonics. *Nat. Photon*. **5**, 397–405 (2011).

[14] Hausmann, B. J. M., Bulu, I., Venkataraman, V., Deotare, P. & Lončar, M. Diamond nonlinear photonics. *Nat. Photon*. **8**, 369–374 (2014).

[15] Trojánek, F., Žídek, K., Dzurňák, B., Kozák. M. & Malý, P. Nonlinear optical properties of nanocrystalline diamond. *Opt. Exp.* **18**, 1349−1357 (2010).

[16] Almeida, J. M. P. *et al*. Nonlinear optical spectrum of diamond at femtosecond regime. *Sci. Rep*. **7**, 14320 (2017).

[17] Sipahigil, A. *et al.* Indistinguishable photons from separated silicon-vacancy centers in diamond. *Phys. Rev. Lett*. **113**, 113602 (2014).

[18] Umeda, T. *et al.* Negatively charged boron vacancy center in diamond. *Phys. Rev. B* **105**, 165201 (2022).
